# Supplementary material for: Solar Light Photoactive Floating Polyaniline/TiO2 Composites for Water Remediation
Source: Nanomaterials (Basel). 2021 Nov 15;11(11):3071. doi: 10.3390/nano11113071 (PMC8619583; doi:10.3390/nano11113071)
Supplement: Supplementary file 1 [file nanomaterials-11-03071-s001.zip › nanomaterials-1443545-supplementary.pdf]

nanomaterials

Supporting Information

Solar Light Photoactive Floating Polyaniline/TiO2 Composites for Water Remediation

Ermelinda Falletta [1,2,*], Anna Bruni [1], Marta Sartirana [1], Daria C. Boffito [3], Giuseppina Cerrato [4], Alessia Giordana [4], Ridha Djellabi [1,2], Erfan S. Khatibi [1] and Claudia L. Bianchi [1,2]

1 Department of Chemistry, Università degli Studi di Milano, via Golgi 19, 20133 Milano, Italy; anna.bruni@unimi.it (A.B.); marta.sartirana@unimi.it (M.S.); ridha.djellabi@unimi.it (R.D.); erfan.saberkhatibi@studenti.unimi.it (E.S.K.); claudia.bianchi@unimi.it (C.L.B.)
2 Consorzio Interuniversitario Nazionale per la Scienza e Tecnologia dei Materiali (INSTM), via Giusti 9, 50121 Florence, Italy
3 Polytechnique Montréal—Génie Chimique 2900 Boul, Edouard Montpetit—H3T 1J4, Montréal, QC H3C 3A7 , Canada; daria-camilla.boffito@polymtl.ca
4 Department of Chemistry, Università degli Studi di Torino, via Pietro Giuria, 7, 10125 Torino, Italy; giuseppina.cerrato@unito.it (G.C.); alessia.giordana@unito.it (A.G.)
* Correspondence: ermelinda.falletta@unimi.it; Tel.: +39-025-031-4410

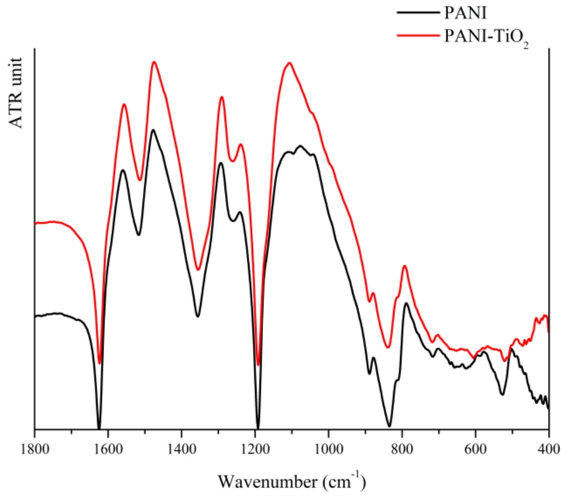

Figure S1. ATR-FTIR spectra of PANI and PANI/TiO_2.

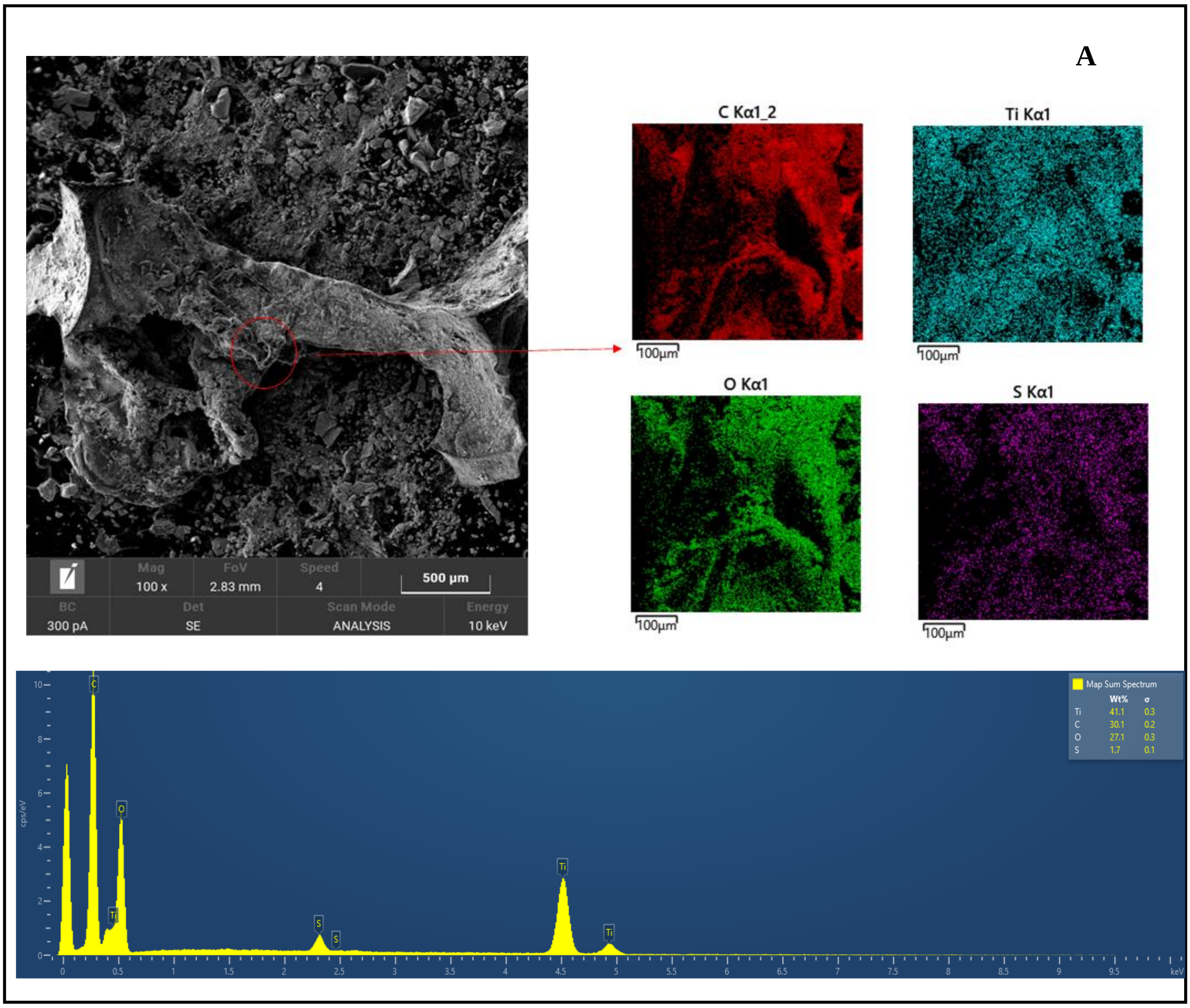
A
Mag
100 x
FoV
2.83 mm
Speed
4
500 µm
BC
300 pA
Det
SE
Scan Mode
ANALYSIS
Energy
10 keV
C Kα1_2
Ti Kα1
O Kα1
S Kα1
100µm
Map Sum Spectrum
Wt% σ
Ti 41.1 0.3
C 30.1 0.2
O 27.1 0.3
S 1.7 0.1
cps/eV
keV

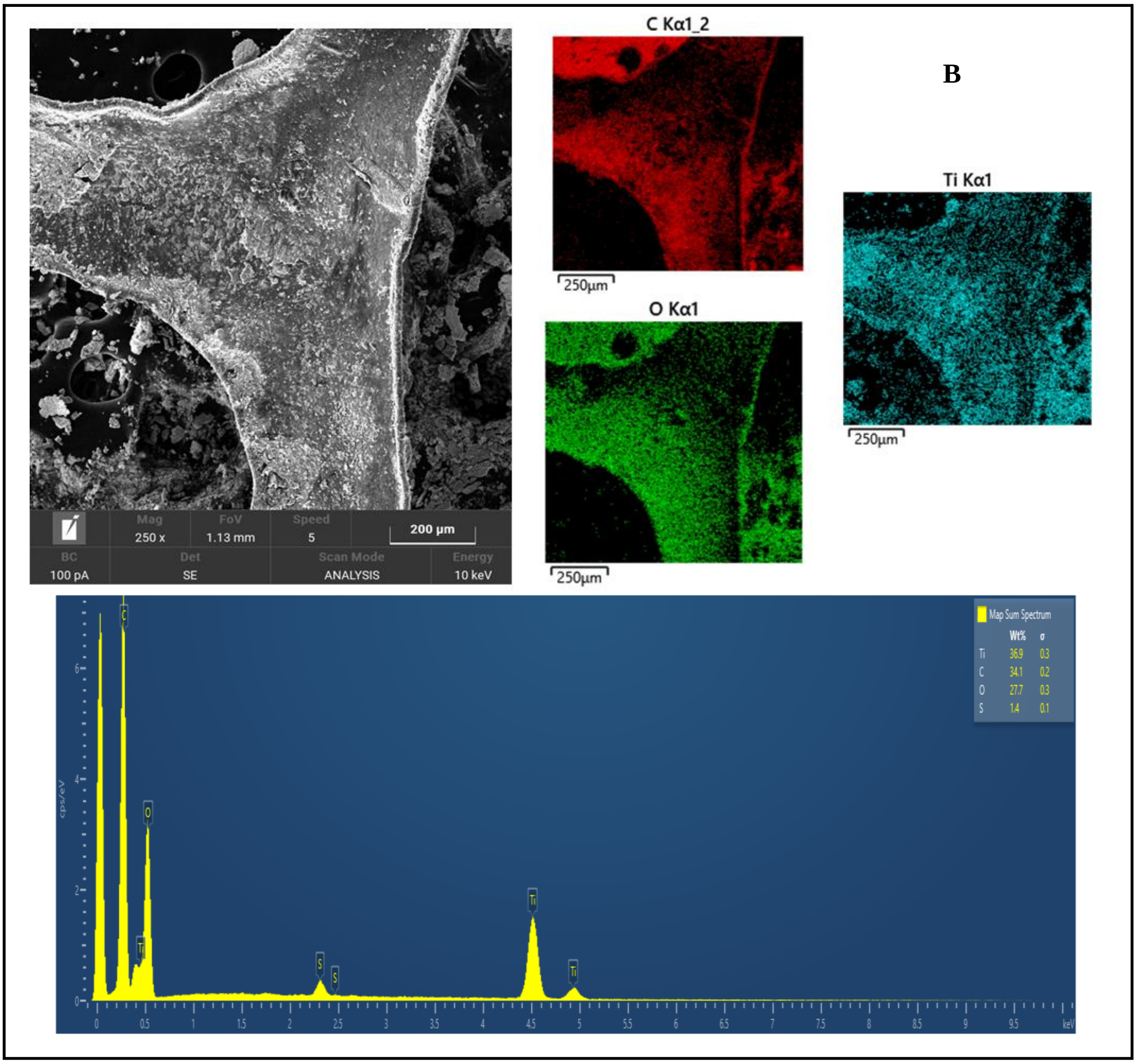

Figure S2. FESEM images and EDS analyses of (**A**) PT/PU-4 and (**B**) PT/PU-6.

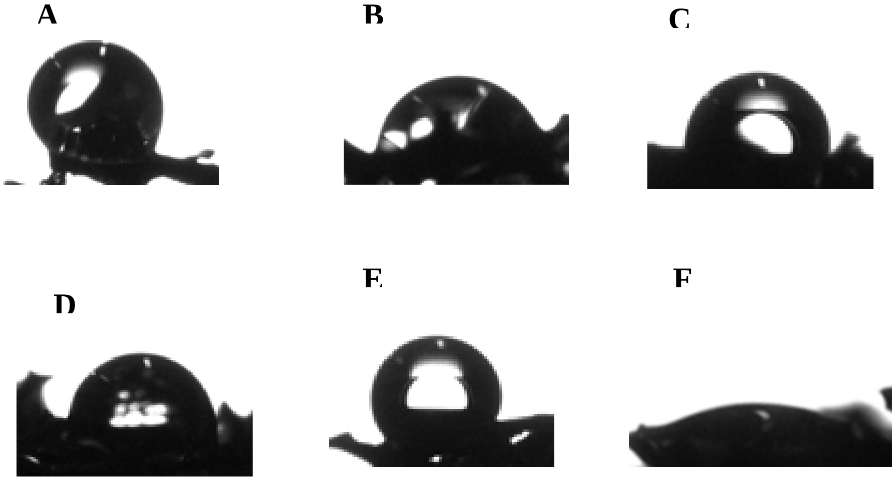

Figure S3. Contact angle images for droplets water on: (**A**) P/PU, (**B**) PT/PU-1, (**C**) PT/PU-2, (**D**) PT/PU-3, (**E**) PT/PU-4, (**F**) PT/PU-5.

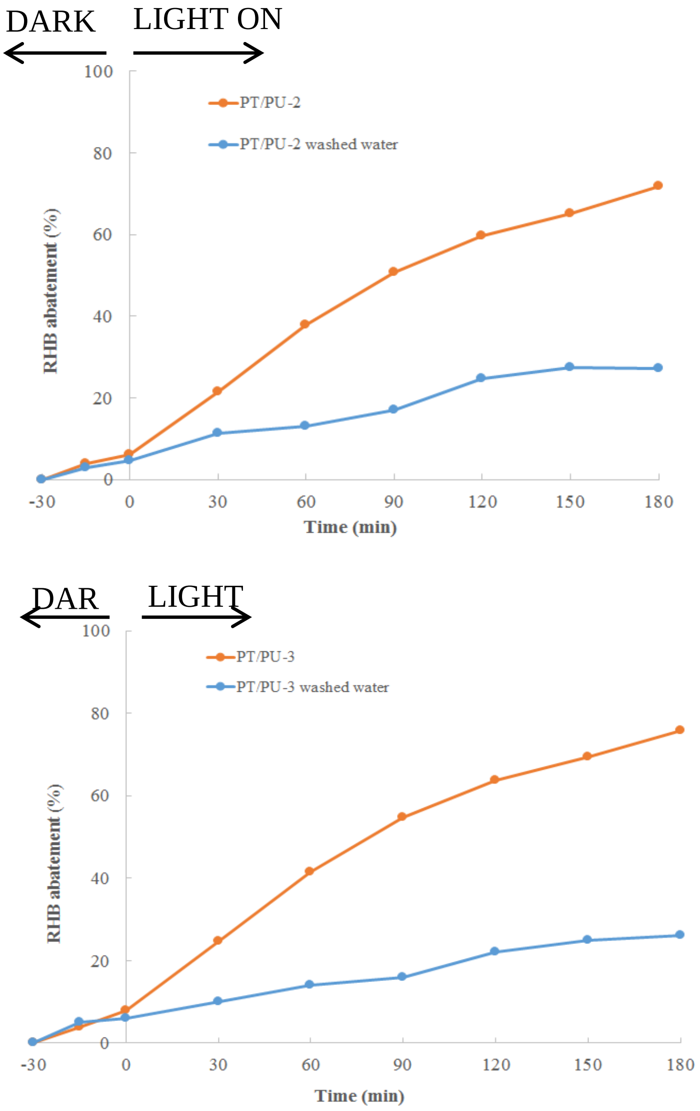

Figure S4. Reuse of PT/PU-2 (up) ad -3 (down) for RHB abatement after washing with water
